# Strategic Risk Based Forecasting of Brent Crude Oil Prices: A Comparative Analysis of Econometric and Machine Learning Models

**DOI:** 10.3390/e28050539

**Published:** 2026-05-09

**Authors:** Tuğçe Ekiz Yılmaz, Cemal Zehir

**Affiliations:** 1 Department of Business Administration, Yildiz Technical University, Davutpaşa Campus, Istanbul 34220, Türkiye; czehir@yildiz.edu.tr; 2 Eurasian Economic International Scientific Research Center, Azerbaijan State University of Economics, Baku 1001, Azerbaijan

**Keywords:** Brent crude oil price forecasting, strategic risk, geopolitical risk index (GPR), CBOE volatility index (VIX), U.S. 10-year Treasury yield (DGS10), ARIMAX, XGBoost, random forest, LightGBM, machine learning

## Abstract

Brent crude oil prices are strategically important due to their sensitivity to geopolitical developments, financial market stress, and global monetary conditions. This study examines whether strategic risk indicators improve the forecasting performance of Brent crude oil returns within an integrated econometric and machine learning framework. Monthly data from January 2001 to December 2025 are employed, using the Global Geopolitical Risk Index (GPR), the CBOE Volatility Index (VIX), and the U.S. 10-year Treasury yield (DGS10) as key explanatory variables. Methodologically, the analysis first estimates benchmark econometric models, including ARIMAX (AutoRegressive Integrated Moving Average with Explanatory Variable) and ARIMAX-gjrGARCH (Glosten-Jagannathan-Runkle Generalized Autoregressive Conditional Heteroscedasticity, and then implements machine learning models, namely XGBoost (eXtreme Gradient Boosting), LightGBM (Light Gradient Boosting Machine), and Random Forest, to capture potential nonlinear relationships. Using sMAPE (Symmetric Mean Absolute Percentage Error), forecast performance is assessed over multiple forecast horizons under a rolling-origin framework. Across several forecasting horizons and train-test split configurations, the empirical results consistently show that machine learning techniques, especially LightGBM, offer superior out-of-sample forecasting accuracy. These findings suggest that the dynamics of Brent crude oil returns are influenced by complex and nonlinear relationships between macro-financial conditions, financial uncertainty, and geopolitical risk. The study concludes that flexible data-driven forecasting frameworks offer stronger predictive performance than benchmark econometric models under strategic risk conditions and provide useful implications for energy market risk management and policy decision-making.

## 1. Introduction

Crude oil prices represent a crucial strategic indicator for global economic activity, financial markets, and energy policy. Fluctuations in oil prices directly impact inflation, economic growth, trade balances, and financial stability; therefore, the demand for reliable forecasting models has been consistently increasing. Preliminary research has shown that oil price series exhibit stylized characteristics similar to those of financial time series, such as high volatility, heavy-tailed distributions, and volatility clustering [1]. Additionally, structural breaks and regime shifts make it difficult to fully capture the oil market dynamics through linear and constant-parameter models [2].

This complex structure has resulted in the development of decomposition, hybrid modeling, and ensemble learning methodologies in the literature. Models utilizing variational mode decomposition, independent component analysis, and grey wave techniques aim to capture the multi-component characteristics of oil price series [3,4,5]. Similarly, quadratic–residual fusion methods [6], intraday hybrid approaches [7], and ensemble learning techniques [8,9] represent significant methodological advances aimed to enhance forecasting performance.

In recent years, machine learning (ML) and deep learning (DL) methods have gained significant prominence in the literature on oil price forecasting. Research examining the performance of classical ML models show that nonlinear relationships are more efficiently captured by ML algorithms [10]. XGBoost (Extreme Gradient Boosting) methodologies and tree-based ensemble models have demonstrated superior predictive performance in oil price forecasting [11,12,13]. Deep learning architectures, on the other hand, have achieved extensive adoption due to their capacity to model intricate temporal dependencies [14,15,16].

The increasing application of ML methods extends beyond oil prices alone. Applications including gold price forecasting [17], stock market prediction [18,19] and asset pricing under financial uncertainty [20] show the widespread utilization of these methodologies throughout the financial system. In the energy sector, ML applications have expanded to areas including emission forecasting [21], oil consumption prediction [22], industrial process modeling [23], and infrastructure risk analysis [24,25].

Indicators of uncertainty and risk play a pivotal role in the oil price literature. Derivatives market information and volatility indicators are critical factors influencing oil price volatility [26,27]. The effects of geopolitical risk, economic policy uncertainty, and financial market stress indicators on oil prices and volatility have been extensively examined in the literature [28,29,30,31,32]. Geopolitical shocks such as wars have been demonstrated to affect model performance [33]. The integration between the oil market and the financial system, as well as the dynamics of volatility and risk transmission mechanisms, has received considerable interest in previous studies [34,35,36,37,38,39,40].

Nevertheless, a review of the literature reveals that the majority of studies either concentrate only on machine learning methods, depend solely on econometric models, or the others are constrained to unidimensional uncertainty indicators. Research that simultaneously examines multidimensional strategic risk indicators—such as financial market uncertainty (VIX), geopolitical risk (GPR), and the macro-financial interest rate channel (DGS10)—and assesses their contribution to Brent crude oil price forecasting within both econometric and machine learning frameworks is limited. This perspective is consistent with the structural oil market literature, which emphasizes that not all oil price shocks have the same economic interpretation and may originate from distinct sources [41]. In this study, the term strategic risk refers to a set of external risk factors that jointly influence oil market dynamics. Specifically, it captures three distinct but related dimensions: geopolitical risk, financial market uncertainty, and macro-financial conditions, whose dimensions are operationalized through the Global Geopolitical Risk Index (GPR), the CBOE Volatility Index (VIX), and the U.S. 10-year Treasury yield (DGS10), respectively. This clarification aims to improve conceptual transparency and avoid potential ambiguity.

Accordingly, strategic risk is not treated as a single homogeneous factor but as a structured representation of multiple sources of uncertainty affecting oil return behavior.

Alongside traditional volatility-based risk indicators, the notion of entropy offers a theoretical framework for measuring uncertainty, disorder, and information content in financial and energy markets. From an information-theoretic perspective, the evolution of oil prices in response to geopolitical and financial shocks can be seen as a system defined by fluctuating information complexity and the transmission of uncertainty. Periods of higher uncertainty can therefore correlate with increased entropy in market signals, indicating a more chaotic and less anticipated return-generating mechanism.

This study aims to address this gap. Monthly data from the post-2001 period are utilized to forecast Brent crude oil prices based on financial, geopolitical, and macroeconomic risk indicators in both econometric and machine learning frameworks. Specifically, an ARIMAX model and a set of tree-based machine learning models—including XGBoost, Light Gradient Boosting Machine (LightGBM), and random forest—were employed to provide a comparative assessment of predictive performance. In doing so, the study seeks to provide both analytical and empirical contributions to the field on strategic risk-based oil price forecasting.

## 2. Literature Review

Forecasting crude oil prices has historically been a subject of substantial interest in energy economics and finance; however, the discipline has undergone a notable methodological and theoretical transformation in recent years. Initial empirical research demonstrated that oil price series display stylized characteristics of financial time series. Evidence from the Brent oil market indicates that price distributions are heavy-tailed, volatility clustering occurs, and responses to shocks are asymmetric [1]. A heavy-tailed distribution implies that price fluctuations produce extreme values more frequently than anticipated under a normal distribution; in other words, significant price increases and decreases occur with higher probability. These characteristics signify that the market is highly sensitive to sudden shocks and that extreme fluctuations have a systematic quality. Furthermore, oil prices demonstrate structural breaks, and regime shifts over time, suggesting that linear and constant-parameter models may inadequately represent these complex dynamics [2,42].

This complex structure has fostered the advancement of decomposition and hybrid modeling methodologies in the literature. The strategy of decomposing time series into several frequency components and forecasting each component with an appropriate model has been demonstrated to improve predictive accuracy. Notable examples include studies employing variational mode decomposition and independent component analysis [3], grey wave-based multi-step forecasting models [5], and quadratic–residual fusion approaches [6]. Similarly, hybrid models intraday data [7] and ensemble approaches [9] support the multi-component characteristics of oil price dynamics. Recent approaches based on independent component analysis seek to improve modeling precision by decomposing the oil price formation mechanism into its fundamental components [43]. Furthermore, integrating fractal and multifractal characteristics into machine learning models has surfaced as a novel approach to more accurately present the complex multi-scale dynamics of time series and enhance forecasting performance [44].

The application of ML and DL algorithms in oil price forecasting has significantly increased in recent years. Comparative analyses of classical ML models [10] indicate that nonlinear relationships can be captured more effectively by ML methods. XGBoost methodologies have become particularly prominent in the oil price forecasting literature [11,12,13]. The use of search engine data into forecasting models has also introduced a new aspect to the literature [45]. Deep learning architectures have achieved widespread utilization due to their capacity to capture complex temporal dependencies [14,15,16]. Ensemble learning approaches enhance predictive accuracy by integrating model diversity [8,9]. Hybrid ARIMAX-LSTM frameworks further improve forecasting accuracy by jointly modeling linear and nonlinear components [46]. Meanwhile, early artificial neural network-based methods constitute the forerunners of ML applications in oil price forecasting. ANFIS-based forecasting studies exemplify the historical development of artificial intelligence-driven methods in oil price modeling [47].

The increasing popularity of ML approaches has not been attributed to oil price forecasting but has expanded across a wide broader set of applications within finance and energy. In financial markets, ML methods have generated a substantial body of literature, encompassing gold price prediction [17], modeling stock market price fluctuations [18,19], and analyzing asset price dynamics under financial uncertainty [20]. These advancements indicate that the complex and nonlinear structure of financial asset pricing can be more accurately represented by data-driven learning algorithms. In the energy sector, ML applications have diversified beyond price forecasting to include the multidimensional nature of energy systems. Forecasting emission levels [21], projecting of oil consumption [22], modeling of industrial processes [23], and conducting risk analyses for critical infrastructure [24] all reflect this expansion trend. Collectively, these studies highlight the strong capability of ML techniques to analyze energy systems characterized by uncertainty, complexity, and interdependence [25]. Recent research has further extended the literature to include the behavioral dimension by examining the impact of investor sentiment and expectation indicators on oil returns using an ML framework [48].

In the literature on oil price, indications of uncertainty and risk have been an important area of research. Information from the derivatives market and volatility indicators significantly influence oil price volatility [26,27]. Research indicates that oil price volatility provides greater informational value than the price level in macroeconomic forecasting [49]. Oil price uncertainty has also been found to extend beyond commodity markets into financial markets, significantly impacting stock returns and market risk perceptions; this highlights the importance of transmission mechanisms by which energy market shocks propagate across the broader financial system [50]. The impact of geopolitical risk on oil volatility has been analyzed using both econometric and ML approaches [28,29]. The time–frequency effects of VIX, GPR, and EPU shocks [30,51], as well as news-based uncertainty indicators [31,32], play a decisive role in oil markets during periods of crisis. The incorporation of geopolitical shocks such as wars into forecasting models has also been shown to have an impact on predictive accuracy [33].

The integration of the oil market with the financial system along with the transmission of volatility have been extensively documented in the literature. Studies investigating return and volatility spillovers between Chinese and international crude oil futures markets indicate bidirectional risk transmission mechanisms across global and regional oil markets, highlighting an increasing financialization of energy markets [34]. Similarly, research analyzing volatility spillovers from the oil market to the Chinese stock market shows that connections between financial and commodity markets have strengthened through WTI, Brent, and implied volatility indicators [35]. Moreover, other studies addressing the dynamic interrelations among geopolitical risk, economic policy uncertainty, and market volatility demonstrate that these risk channels generate simultaneous fluctuations in both commodity and equity markets, with risk spillovers accelerating notably during crisis periods [36]. Asymmetric analyses on European sectors further reveal heterogeneous risk transmission within the financial system, indicating that economic policy uncertainty, geopolitical risk, and market sentiment influence regional equity markets with differing directions and magnitudes [37]. In addition, the role of China on global oil prices has been examined through macroeconomic demand and geoeconomic connections [40]. Likewise, the short-term forecasting relationship between crude oil prices and petroleum product prices offers more evidence of market interdependence [52]. Moreover, studies examining the correlation between petroleum product prices and crude oil prices show that price transmission mechanisms function through refinery margins, demand structure, and market expectations, and that this relationship possesses short-term predictive capability [53].

Finally, the research on volatility forecasting for energy commodities increasingly compares ML and econometric models. Evidence from comparisons between generalized autoregressive conditional heteroskedasticity (GARCH) models and support vector regression (SVR) indicates that ML techniques are competitive in volatility modeling [54]. Furthermore, studies on market crash prediction [55] and the classification of risk characteristics [56] illustrate the effectiveness of ML in identifying financial risk occurrences.

Overall, this comprehensive body of literature underlines the importance of ML and hybrid approaches in oil price forecasting, highlights the critical role of uncertainty indicators, and stresses the necessity of considering connections with the financial system. However, studies that simultaneously utilize financial uncertainty indicators (VIX), the Geopolitical Risk Index (GPR), and the macro-financial interest rate indicator (DGS10) to comparatively forecast Brent crude oil prices through both econometric and ML models remain limited. This study aims to fill this deficiency and contribute to the existing literature.

## 3. Data and Methodology

This study aims to forecast Brent crude oil prices using strategic risk indicators and examines statistical time series modeling and machine learning-based approaches within a comparative framework. The analysis was conducted using a dataset constructed at a monthly frequency covering the period from January 2001 to December 2025, yielding a total of 300 observations. The methodological framework was designed to capture the nonlinear, multi-component, and high-uncertainty dynamics of energy markets. Missing observations within each month were handled during aggregation by excluding unavailable values (na.rm = TRUE). No interpolation or data imputation procedure was applied to preserve the stochastic properties of the financial time series.

The monthly logarithmic return of Brent crude oil prices was employed as the dependent variable. Daily Brent spot prices were obtained from the Federal Reserve Economic Data (FRED) database (Federal Reserve Bank of St. Louis, St. Louis, MO, USA) and converted into monthly averages [57]. This aggregation ensures temporal alignment with strategic risk indicators and reduces excessive high-frequency noise. Accordingly, the monthly logarithmic return is calculated as follows:

(1) $$r_{t}=\;100\;\times \left[\ln\left(P_{t}\right)-ln(P_{t-1})\right]$$

where:

- $r_{t}$ denotes the monthly return of Brent crude oil prices at time *t* (in percent),
- $P_{t}$ represents the monthly average price of Brent crude oil at time *t*,
- $P_{t-1}$ denotes the Brent price in the previous month, and
- ln is the natural logarithm operator.

This transformation is preferred to achieve stationarity, eliminate scale differences, and measure percentage changes symmetrically while mitigating the impact of excess variance.

The model framework is constructed on three primary strategic risk channels identified in the literature as key drivers of the oil market. Geopolitical risk is measured using the Global Geopolitical Risk Index (GPR) developed by [58]. The GPR variable is used in its original index form as provided by the source dataset. Given that the index may exhibit non-stationary behavior in levels during certain sub-periods, standard unit root tests (ADF and PP) were conducted. To ensure consistency with the forecasting design and to mitigate potential non-stationarity concerns, the variable was incorporated in lagged form within the empirical models. Financial market uncertainty was proxied by the CBOE Volatility Index (VIX), a widely used indicator of market volatility [59]. Monetary policy and macro-financial conditions were incorporated via the U.S. 10-year Treasury yield (DGS10) [60]. A key consideration in the data construction was the alignment of data frequencies across variables. The GPR index is inherently available at a monthly frequency, whereas VIX and DGS10 are originally observed at a daily frequency. To ensure temporal consistency and avoid mixed-frequency biases in the forecasting framework, all variables were aggregated to a common monthly frequency. Specifically, daily observations of VIX and DGS10 are converted into monthly averages. This aggregation allows for a coherent integration of macro-financial and geopolitical indicators within a unified forecasting structure. While monthly aggregation helps reduce high-frequency noise and ensures consistency across variables, it may also smooth short-lived market fluctuations and abrupt changes in financial or geopolitical conditions. This trade-off is acknowledged as a limitation of the data construction process.

The dataset was divided chronologically into training and testing subsets, where 80% of the observations were used for model estimation and the remaining 20% were reserved for out-of-sample forecast evaluation. This design preserves the temporal ordering of the data and prevents look-ahead bias. In addition, to assess the robustness of the empirical findings, alternatives of both 75-25% and 70-30% training-testing splits were also considered. This robustness check makes it possible to evaluate whether the comparative forecasting results remain stable under a different sample partition.

Two different modeling approaches were employed in this study to evaluate the predictive role of strategic risk indicators in Brent oil returns. First, the ARIMAX model is utilized to jointly examine the autoregressive structure of oil returns and the contemporaneous effects of strategic risk variables. This specification serves as the benchmark linear framework, capturing autocorrelation dynamics alongside the linear influence of exogenous risk factors. In addition to the baseline ARIMAX specification, the adequacy of the model was further evaluated through residual diagnostics. The presence of volatility clustering in the residuals motivated the extension of the model to incorporate conditional heteroskedasticity. Accordingly, the ARIMAX model was augmented with a GARCH structure, as residual diagnostics provide strong evidence of autoregressive conditional heteroskedasticity (ARCH) effects, suggesting that the assumption of constant variance is violated and that volatility evolves over time. In this study, the GARCH component was incorporated to explicitly model the conditional variance dynamics of Brent crude oil returns, which exhibit well-known stylized facts such as volatility clustering and time-varying uncertainty. Financial time series, particularly commodity returns, are characterized by periods of high and low volatility that cannot be adequately captured under the assumption of constant variance.

Accordingly, the ARIMAX-GARCH specification extends the baseline ARIMAX model by allowing the variance of the error term to evolve over time. This enables the model to capture the persistence of shocks and the clustering behavior observed in oil market volatility. The inclusion of the GARCH structure is therefore motivated by the need to model risk dynamics and uncertainty propagation rather than to directly improve point forecasts of the conditional mean. While the mean equation remains identical to the ARIMAX formulation, the GARCH model is estimated on the residuals of the ARIMAX mean equation, such that the conditional variance is modeled separately without altering the mean forecast structure. The variance equation is specified as:

(2) $$\sigma_{t}^{2}=\omega +\alpha \varepsilon_{t-1}^{2}+\beta \sigma_{t-1}^{2}$$

where:

- $\sigma_{t}^{2}$ denotes the conditional variance at time *t* (in percent),
- $\varepsilon_{t-1}^{2}$ represents the lagged squared residual,
- $\sigma_{t-1}^{2}$ is the lagged conditional variance,
- ω is the constant term representing the long-run variance level,
- α is the ARCH parameter capturing the short-term impact of past shocks,
- β is the GARCH parameter reflecting the persistence of volatility through lagged conditional variance.

The parameter α measures how strongly new shocks affect current volatility, whereas β captures the degree to which past volatility is transmitted over time. A high value of (α+β) indicates strong volatility persistence, which is a common characteristic of financial time series. For model stability and stationarity of the variance process, the condition α+β<1 must hold.

Second, machine learning algorithms are implemented to model potential nonlinearities and interaction effects in the oil market. These methods approximate a flexible nonlinear mapping of the form:

(3) $${\hat{y}}_{t}=\;\sum_{m=1}^{M}f_{m}\left(x_{t}\right)$$

where:

- ${\hat{y}}_{t}$ denotes the predicted value of Brent oil returns at time *t*,
- M the total number of decision trees in the ensemble,
- $f_{m}\left(\cdot \right)$ denotes *m*-th decision tree learner, and,
- $x_{t}$ is the feature vector used to predict Brent oil returns at time *t*, constructed using only information available up to time *t* − 1, including lagged returns and lagged exogenous risk indicators.

### 3.1. Random Forest (Bagging Framework)

Random forest [61] represents the bagging paradigm and constructs multiple decorrelated decision trees using bootstrap resampling and random feature selection. Final predictions are obtained via aggregation across trees, which primarily reduces variance and enhances generalization performance, particularly in noisy financial environments.

### 3.2. Gradient Boosting Family: XGBoost and LightGBM

In contrast, XGBoost [62] and LightGBM [63] belong to the gradient boosting family, where trees are built sequentially to minimize a predefined loss function. Boosting methods iteratively refine the errors of preceding learners, thereby focusing on bias reduction and enabling the capture of complex nonlinear interactions. XGBoost incorporates explicit regularization to control model complexity, whereas LightGBM employs a leaf-wise tree growth strategy that improves computational efficiency in high-dimensional settings.

To facilitate the application of tree-based algorithms that cannot directly process raw time-series observations, the forecasting problem is reformulated into a supervised learning framework. Within this framework, two primary sources of information are provided to the model:

- Autocorrelation Dynamics (Lag Features): Past values of Brent oil returns are included as input features in the machine learning models using eight lagged observations ${(r}_{t-1},r_{t-2},\ldots ,r_{t-8})$. These lagged features capture the internal dynamics of the series, short-term memory, momentum effects, and potential persistence patterns in oil returns. This structure enables the models to learn both recent fluctuations and medium-term temporal dependencies. The selection of lagged return variables is informed by the autocorrelation. The PACF function exhibits a dominant spike at lag 1, followed by rapidly diminishing partial autocorrelations across higher lags, indicating a short-memory process. However, to allow the machine learning models to capture potential medium-term dependencies and nonlinear temporal interactions, a lag length of eight periods is adopted. This choice balances model flexibility and dimensionality, avoiding excessive feature expansion while retaining sufficient temporal information. In addition, this lag structure is validated through the forecasting performance of the models under a rolling-origin evaluation framework.
- Exogenous Strategic Risk Indicators: GPR, VIX, and DGS10 are incorporated into the model in lagged form. Specifically, one-period lagged values of these variables (${GPR}_{t-1}$, ${VIX}_{t-1}$ and ${DGS10}_{t-1}$) are used as predictors to ensure a genuine forecasting framework and avoid contemporaneous information leakage. These indicators reflect geopolitical shocks, financial market uncertainty, and macro-financial conditions affecting the oil market.
- Entropy-Based Information Measure: In addition to the conventional strategic risk indicators, an entropy-based variable is incorporated to capture the informational complexity and uncertainty dynamics of the oil market. Specifically, a rolling Shannon entropy measure is computed from Brent oil returns using a moving window approach. This variable reflects the degree of disorder and unpredictability in the return-generating process, providing a complementary perspective to volatility-based and macro-financial risk indicators. The entropy measure is included in the model as a one-period lagged predictor (*Entropy*_*t*−1_) ensuring consistency with the forecasting framework and avoiding contemporaneous information leakage.

Through this structure, the machine learning models estimate the following general nonlinear relationship:

(4) $$r_{t}=\;f\left(r_{t-1},r_{t-2},\ldots ,r_{t-8},{GPR}_{t-1},{VIX}_{t-1},{DGS10}_{t-1},\;{Entropy}_{t-1}\right)$$

The symbols used in the model are defined as follows:

- $r_{t}$: Monthly logarithmic return of Brent crude oil at time *t*,
- $r_{t-1},r_{t-2},\ldots ,r_{t-8}$: Brent return values lagged by 1 through 8 months, respectively,
- ${GPR}_{t-1}$: Global Geopolitical Risk Index at time *t* − 1,
- ${VIX}_{t-1}$: Financial Market Volatility Indicator at time *t* − 1,
- ${DGS10}_{t-1}$: US 10-year Treasury yield at time *t* − 1,
- ${Entropy}_{t-1}$: Rolling Shannon entropy of Brent oil returns at time *t* − 1,
- t: Monthly time index.

To ensure a genuine forecasting framework and avoid any contemporaneous information leakage, all explanatory variables were incorporated in lagged form. Specifically, GPR, VIX, and DGS10 were used as one-period lagged predictors when forecasting Brent oil returns at time *t*.

Accordingly, only information available at time *t* − 1 was used for prediction, ensuring that the empirical design corresponds to a true out-of-sample forecasting exercise rather than a nowcasting framework.

In this context, $f\left(\cdot \right)$ is not confined to a linear functional form; instead, it represents nonlinear relationships and interactions among variables via decision trees. This approach goes beyond the linear assumptions of the ARIMAX model and offers the potential to capture the intricate and regime-dependent dynamics of oil returns.

To enhance predictive performance and ensure the robustness of the machine learning framework, all tree-based models are estimated under a grid-search-based hyperparameter optimization procedure. In this context, key model parameters—including tree depth, learning rate, subsampling ratio, node complexity, and feature sampling proportions—are jointly optimized through predefined search grids. Rather than relying on a single arbitrary specification, each parameter is evaluated across low, moderate, and relatively high candidate values in order to capture alternative model complexity levels and regularization strengths. For example, in the XGBoost framework, maximum tree depth, learning rate, subsampling ratio, column sampling ratio, minimum child weight, and gamma parameters are systematically tuned. Similarly, the random forest model is optimized with respect to the number of candidate predictors at each split and minimum node size, whereas LightGBM is tuned over leaf size, learning rate, feature fraction, bagging fraction, and regularization parameters. This structured search strategy allows the identification of parameter combinations that minimize out-of-sample forecasting errors while reducing the risk of overfitting.

Given the temporal structure of the forecasting problem, a rolling-window forecasting framework is employed rather than conventional random validation procedures. Specifically, the models are recursively re-estimated as the estimation window expands over time, and out-of-sample forecasts are generated at each forecast origin. This approach preserves the chronological ordering of the data, eliminates look-ahead bias, and more closely reflects real-world forecasting practice under evolving market dynamics and structural uncertainty.

To evaluate forecasting performance, the dataset is first divided chronologically into 80% training and 20% testing sets, thereby establishing the baseline out-of-sample forecasting framework. As a robustness analysis, all models are additionally re-estimated under alternative 75-25% and 70-30% training-testing splits. Within this rolling-window design, predictive accuracy is assessed across multiple forecasting horizons, namely 1-step, 3-step, 6-step, and 9-step ahead forecasts, in order to examine both short-term predictive precision and longer-horizon forecast stability.

Forecast performance is evaluated using the symmetric mean absolute percentage error (sMAPE), which is preferred due to its scale-independent nature and its suitability for comparing predictive accuracy across different forecast horizons. By normalizing forecast errors relative to the magnitude of the observed and predicted values, sMAPE provides a consistent and comparable measure of forecasting performance, independent of the scale of the underlying series.

Hence, this methodological framework enables the evaluation of strategic risk factors under both linear and nonlinear modeling approaches. While the ARIMAX and ARIMAX-GARCH models serve as benchmark econometric structures capturing linear dependence and volatility dynamics, machine learning models provide a flexible nonlinear forecasting framework capable of modeling complex interactions, regime shifts, and medium-term market behavior. Consequently, the study offers an integrated comparative methodological contribution to the energy price forecasting literature. All empirical analyses, including econometric estimation, machine learning model development, diagnostic testing, hyperparameter optimization, and rolling-window forecasting, were implemented in the R Studio (version 2024.12.1+563) environment using the R programming language.

## 4. Empirical Findings and Discussion

This section presents the forecasting performance of Brent crude oil returns under strategic risk indicators and analyzes the empirical results. First, the descriptive statistics of the variables and the linear relationships among them are examined. Second, stationarity and autocorrelation diagnostics are reported to justify the econometric model specification. Third, residual diagnostic tests are evaluated to assess model adequacy and volatility behavior. Fourth, alternative GARCH specifications are compared. Finally, the out-of-sample forecasting performances of econometric and machine learning models are comparatively discussed.

**Figure 1:**
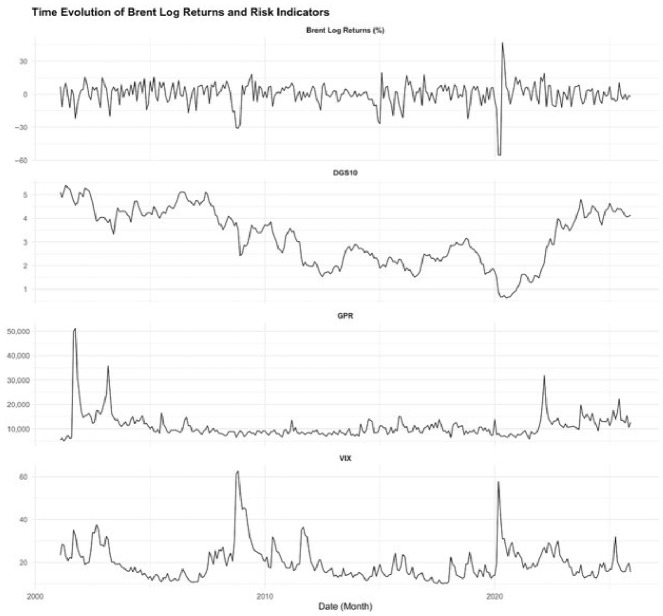
Time evolution of Brent log returns and strategic risk indicators.

Figure 1 depicts the time paths of Brent crude oil log returns after 2001, along with the DGS10, GPR, and VIX series. Brent log returns exhibit significant volatility clustering and abrupt spikes, clearly revealing the oil market’s high uncertainty and sensitivity to shocks. Notably, during the 2008 global financial crisis, dramatic declines in oil returns are observed, accompanied by a considerable increase in the VIX index. Likewise, during the COVID-19 pandemic in 2020, Brent returns experienced an unprecedented negative shock, coinciding with a historically high surge in the VIX. This pattern indicates that the oil market is highly sensitive to financial uncertainty shocks.

The VIX series had a consistently modest and stable pattern during normal periods but exhibited sharp increases during global shock episodes, reflecting sudden changes in risk perceptions in financial markets. Conversely, the GPR series displayed infrequent but more abrupt spikes, representing risk surges that attribute to geopolitical events rather than persistent financial uncertainty. Although increases in GPR were observed during the 2008 and 2020 periods, these movements were not as dramatic as the spikes in the VIX. This difference arises because these incidents were primarily driven by financial and economic shocks rather than direct geopolitical conflicts. This divergence signifies that the oil market responds to different risk channels with varying intensity.

The DGS10 series exhibited a long-term downward trend, particularly reflecting the global transition toward a low-interest-rate regime following the 2008 crisis. This decline persisted throughout the 2010s and reached historically low levels during the 2020 pandemic period. This tendency is consistent with expansionary monetary policies and global liquidity circumstances. Following 2021, however, a considerable increase in interest rates can be observed, signaling a transition toward a monetary tightening policy. Overall, Figure 1 illustrates that oil returns display sudden, asymmetric, and nonlinear reactions during periods of increased uncertainty, clearly revealing the role of financial market uncertainty, geopolitical risks, and macro-financial conditions in oil price dynamics. These observations provide strong empirical support for the limitations of linear models and the necessity of nonlinear modeling approaches.

**Figure 2:**
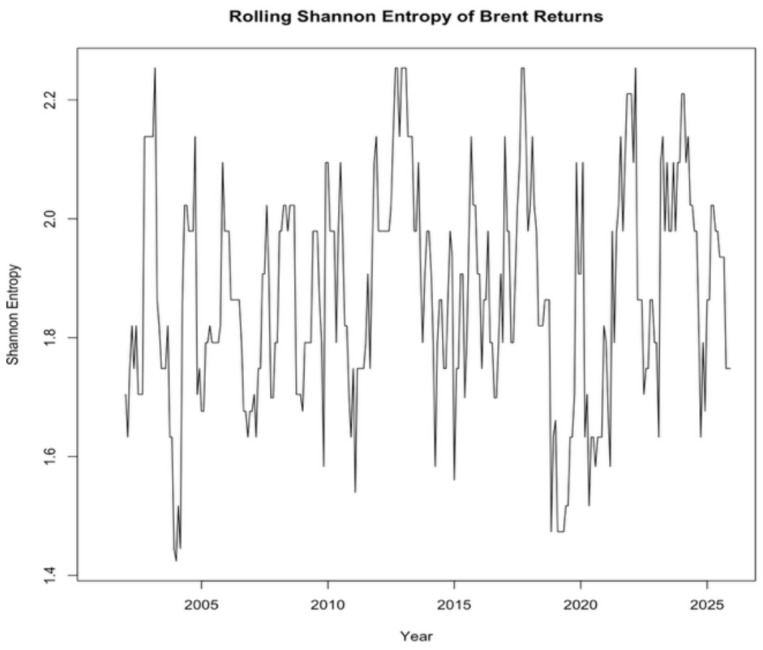
Rolling Shannon entropy of Brent oil returns.

Figure 2 illustrates the time-varying behavior of the rolling Shannon entropy computed from Brent oil returns. The entropy measure exhibited notable fluctuations over the sample period, indicating that the informational complexity and uncertainty structure of the oil market evolve over time rather than remaining constant. Periods associated with major economic and financial disruptions, such as the global financial crisis and the COVID-19 shock, are characterized by elevated entropy levels, reflecting increased unpredictability in return dynamics. This pattern supports the interpretation of entropy as a complementary measure of uncertainty that captures aspects of market complexity beyond conventional volatility indicators. In addition, the correlation between the entropy measure and the absolute value of Brent returns was found to be relatively weak (−0.1505), suggesting that entropy captures informational complexity rather than purely reflecting return volatility.

Table 1 presents the descriptive statistics of the variables used in the analysis. The near-zero mean of Brent crude oil returns indicates that the series fluctuates around its long-term equilibrium. However, the relatively high standard deviation reveals the pronounced volatility structure of the oil market. In particular, the substantially negative minimum value signifies that sharp price declines occurred during certain periods. Since the logarithmic return transformation reflects price increases as positive values and price decreases as negative values, this pattern represents severe price corrections typical of crisis periods. This finding confirms that the oil market is highly sensitive to shocks and characterized by significant unpredictability. The relatively large mean value of the GPR index reflects its original scale, as provided in the source dataset. Since the index is used in its raw form, potential non-stationarity concerns are addressed within the modeling framework through lagged specifications and diagnostic testing.

The extensive value ranges of the GPR and VIX variables show that notable spikes in risk indicators occur during periods of global geopolitical tension and financial uncertainty. In contrast, the DGS10 variable moves within a constrained band, suggesting that interest rates follow a more stable trajectory while still representing an important macro-financial indicator that should be considered in oil market dynamics.

Table 2 presents the results of the augmented Dickey–Fuller (ADF) and Phillips–Perron (PP) unit root tests. The findings indicate that Brent returns, GPR, and VIX are stationary in levels. In contrast, the DGS10 series was found to be non-stationary, as both ADF and PP tests failed to reject the null hypothesis of a unit root. Since the null hypothesis of both ADF and PP tests is that the series contains a unit root, therefore, the first difference of DGS10, denoted by DGS10(∆), was employed in the empirical analysis. The differenced series (DGS10(∆)) was confirmed to be stationary, ensuring the validity of the ARIMAX specification.

**Figure 3:**
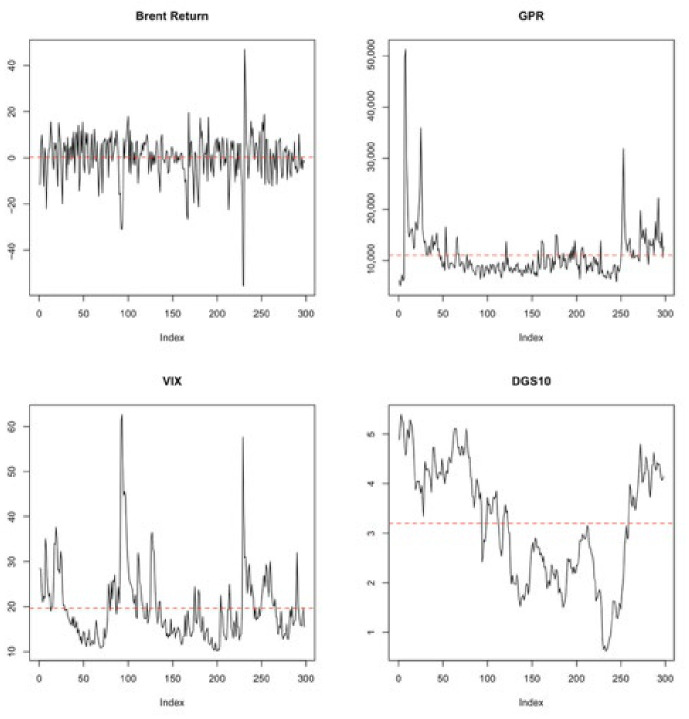
Time series plots of Brent returns and strategic risk indicators (GPR, VIX, and DGS10). The red dashed lines represent the mean values of each series, illustrating their fluctuations around the average level over time.

Figure 3 presents the time series behavior of Brent oil returns and the strategic risk indicators. Brent returns fluctuated around a constant mean with no visible trend, suggesting stationarity. Similarly, the GPR and VIX series exhibited mean-reverting behavior despite occasional volatility spikes. In contrast, the DGS10 series displayed a clear non-stationary pattern with a persistent downward and upward trend over time. This visual evidence is consistent with the unit root test results, which indicate that DGS10 is non-stationary in levels and requires first differencing before inclusion in the model.

As shown in Table 3, the correlation coefficients among the variables were predominantly low. While a negative but relatively weak relationship was observed between Brent crude oil returns and the VIX, which represents financial market uncertainty, the association with the GPR appeared even more limited. The relationship between Brent returns and DGS10, which serves as a proxy for interest rates, was almost negligible. However, it is important to note that low linear correlation does not necessarily imply the presence of nonlinear relationships. Rather, these results suggest that the dependence structure between oil returns and strategic risk indicators may not be adequately captured by simple linear associations. In particular, the effects of risk variables may emerge through nonlinear interactions, threshold effects, or time-varying dynamics that are not reflected in pairwise correlation measures. Such patterns are commonly observed in financial time series, where dependencies often manifest in conditional moments rather than unconditional correlations. Therefore, instead of interpreting low correlations as direct evidence of nonlinearity, these findings are considered as an indication that more flexible modeling approaches may be required to capture the underlying data-generating process. This provides an empirical motivation for employing machine learning models, which are capable of approximating complex nonlinear relationships and interactions among variables.

**Figure 4:**
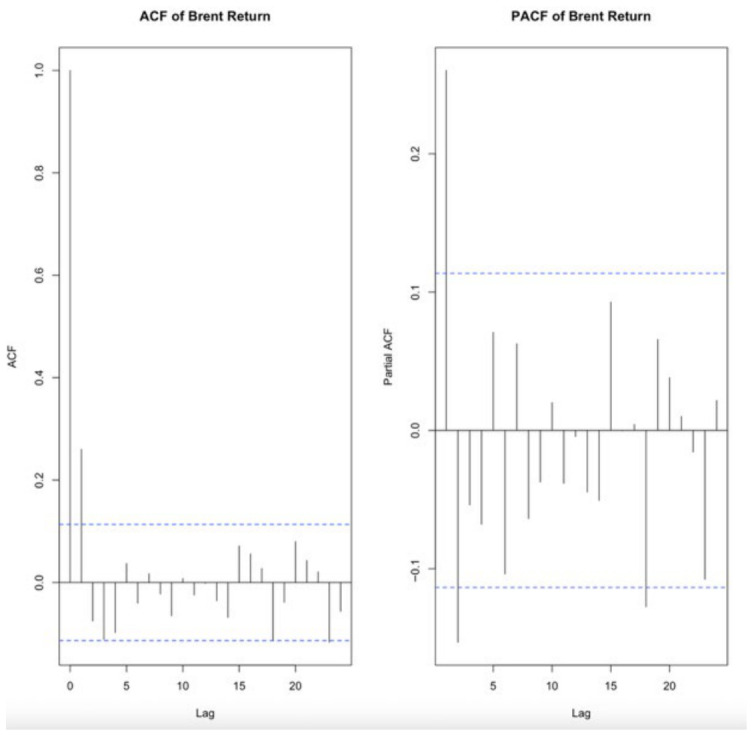
Autocorrelation (ACF) and partial autocorrelation (PACF) functions of Brent oil returns. The blue dashed lines represent the 95% confidence intervals, indicating the statistical significance of the autocorrelations at different lags.

The autocorrelation structure of Brent oil returns was examined using the autocorrelation function (ACF) and partial autocorrelation function (PACF), as seen in Figure 4. The ACF plot revealed a significant spike at the first lag followed by a rapid decay within the confidence bounds, indicating a short-memory process. Similarly, the PACF showed a dominant spike at lag 1 with no persistent structure across higher lags. These findings suggest that the return series does not exhibit strong higher-order autocorrelation, and a low-order moving average structure is sufficient to capture the temporal dependence.

In addition, the optimal ARIMA specification was determined using the “auto.arima” algorithm, which selects an ARIMA(0,0,1) model based on information criteria. This data-driven model selection is fully consistent with the empirical patterns observed in the ACF and PACF diagnostics. Accordingly, an ARIMAX(0,0,1) specification was adopted to model the conditional mean dynamics of Brent oil returns. This consistency between statistical diagnostics and automated model selection enhances the robustness of the model specification.

**Figure 5:**
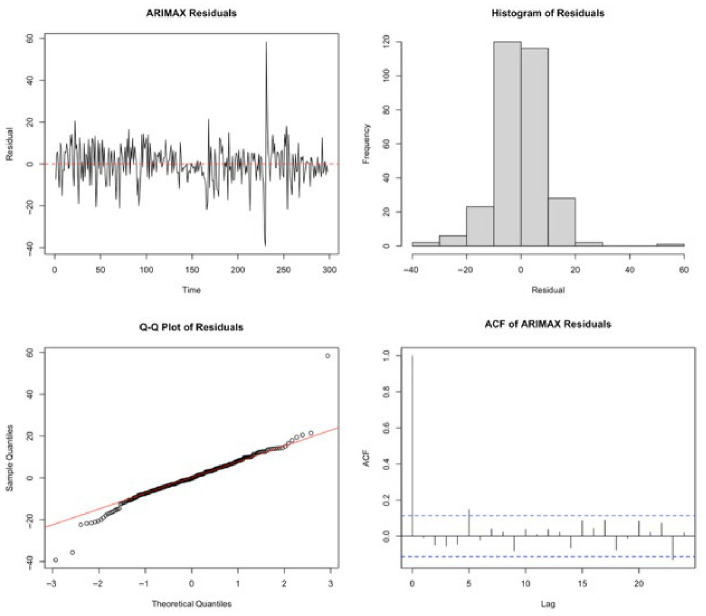
Residual diagnostics of the ARIMAX model.

To evaluate the adequacy of the ARIMAX model, a comprehensive residual diagnostic analysis was conducted. Figure 5 presents the residual time series, histogram, Q–Q plot, and autocorrelation function of the residuals.

The residual series fluctuated around zero without any visible trend, indicating that the conditional mean dynamics were appropriately captured. The histogram and Q-Q plot suggest an approximately symmetric distribution, although slight deviations from normality were observed in the tails, reflecting the presence of extreme values typical in financial time series.

The autocorrelation function of residuals shows that most autocorrelations lay within the confidence bounds, indicating that serial correlation was largely removed. This finding is further supported by the Ljung–Box test results, which failed to reject the null hypothesis of no autocorrelation at conventional significance levels. However, the ARCH LM test strongly rejected the null hypothesis of no ARCH effects, indicating the presence of conditional heteroskedasticity in the residuals. This suggests that while the ARIMAX model adequately captures the mean structure, it fails to model time-varying volatility dynamics. These findings provide strong empirical justification for augmenting the ARIMAX framework with a GARCH-type volatility model.

To formally assess the presence of serial correlation in the residuals, the Ljung–Box test was applied. The test results indicate that the null hypothesis of no autocorrelation was rejected at the 5% significance level $\left(\chi^{2}=13.2584,\;df=7,\;p\text{-}value=0.039\right)$, suggesting that some residual serial dependence remains in the ARIMAX model.

To examine whether the residuals exhibited conditional heteroskedasticity, an ARCH LM test was conducted. The results strongly rejected the null hypothesis of no ARCH effects $\left(\chi^{2}=68.4048,\;df=12,\;p\text{-}value<0.001\right)$, indicating the presence of significant time-varying volatility in the residual series. These findings imply that while the ARIMAX model captures part of the conditional mean dynamics, it is insufficient to fully account for the dependence structure and volatility clustering inherent in oil returns. Therefore, extending the model with a GARCH-type specification is both theoretically and empirically justified.

**Figure 6:**
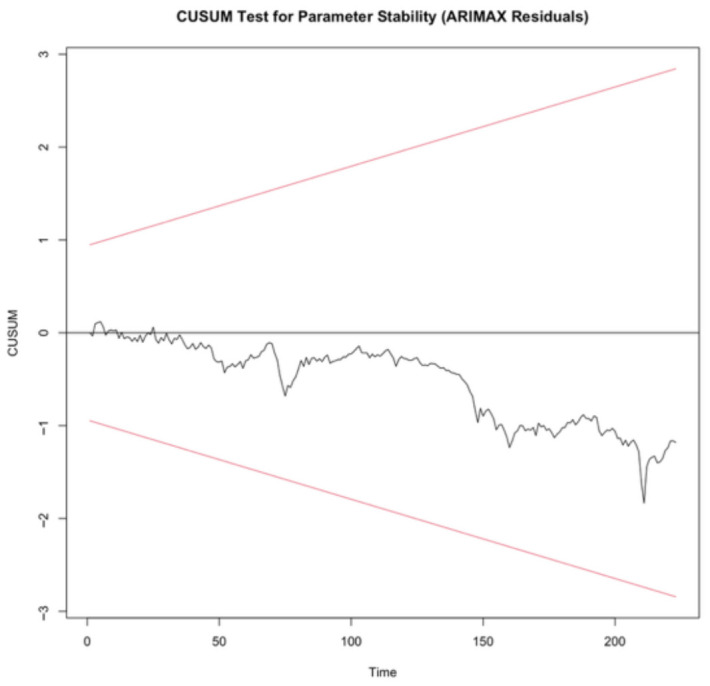
Structural stability analysis using CUSUM tests. The red lines represent the 5% significance boundaries. The model is considered stable as the cumulative sum remains within these critical limits.

**Figure 7:**
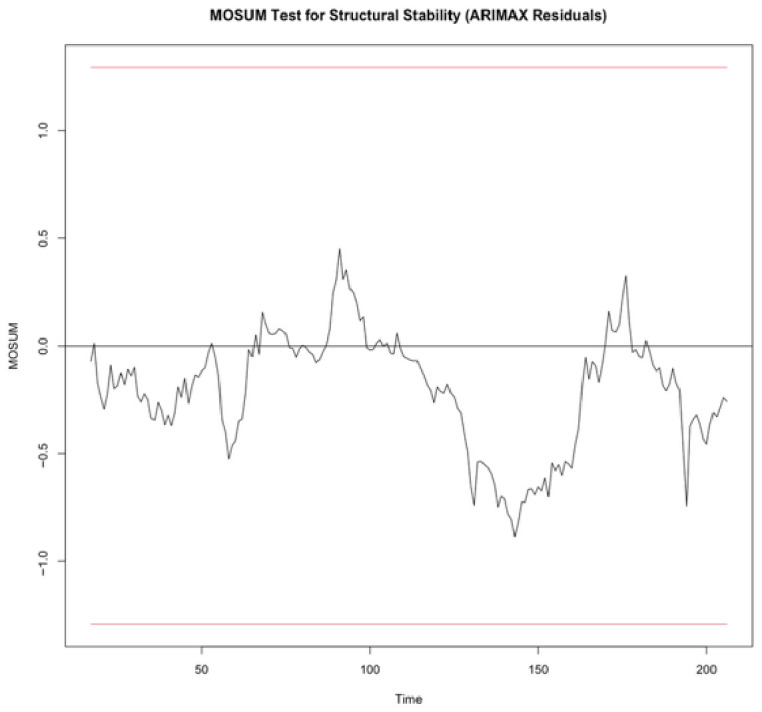
Structural stability analysis using MOSUM tests. The red lines indicate the 5% significance boundaries. The model is considered structurally stable as the test statistic remains within these bounds.

To evaluate the structural stability of the estimated model, the cumulative sum (CUSUM) and moving sum of recursive residuals (MOSUM) tests were employed as pictured in Figure 6 and Figure 7, respectively.

The CUSUM test results indicate that the empirical fluctuation process remained within the critical bounds throughout the sample period, and the null hypothesis of parameter stability cannot be rejected. This finding implies that the model parameters are stable over time and do not exhibit systematic structural changes.

Similarly, the MOSUM test did not show any boundary crossings, indicating the absence of statistically significant localized structural instability. Although some short-term fluctuations were observed, these deviations remained within the confidence bounds and therefore do not provide evidence of structural breaks.

Taken together, the CUSUM and MOSUM results confirm that the model is structurally stable both globally and locally. These findings suggest that the estimated model adequately captures the underlying dynamics of the oil market while remaining robust to short-term variations in geopolitical and financial conditions.

Following the extension of the ARIMAX framework, alternative GARCH-type specifications were estimated and compared based on information criteria. Among the candidate models, the symmetric GARCH specification with a skewed Student’s *t* distribution provided the best fit, indicating the presence of heavy tails and asymmetric behavior in Brent oil return volatility.

This modeling approach is consistent with the broader commodity volatility literature, where more advanced GARCH-type specifications, such as mean-reverting affine GARCH models, have been proposed to better capture the dynamics of commodity prices [64]. However, in the present study, a relatively parsimonious GARCH specification was adopted to maintain comparability with the baseline ARIMAX framework and focus on the primary objective of forecasting performance.

The estimated variance equation parameters confirm the existence of volatility clustering in the series. The ARCH coefficient $\left(\alpha \right)$ captures the immediate impact of new shocks on conditional variance, while the GARCH coefficient $\left(\beta \right)$ reflects the persistence of past volatility over time. The combined magnitude of these parameters suggests moderate volatility persistence, implying that shocks to Brent oil returns have a diminishing yet lasting effect on future volatility. These findings are consistent with the stylized facts of financial and energy market time series, where volatility tends to cluster and persist following periods of market stress, geopolitical shocks, and macroeconomic uncertainty.

To formally present the model selection results, Table 4 reports the comparative performance of alternative GARCH specifications based on the Akaike information criterion (AIC), Bayesian information criterion (BIC), and log-likelihood values. The analysis considered three widely used GARCH-type model families: the symmetric GARCH (sGARCH), exponential GARCH (eGARCH), and Glosten-Jagannathan-Runkle GARCH (gjrGARCH). For each model family, the specification that yielded the lowest AIC value was selected and reported in Table 4.

As presented in Table 4, the gjrGARCH model with a skewed Student’s *t* distribution yielded the lowest AIC and BIC values, along with the highest log-likelihood, indicating superior model fit among the competing alternatives. This finding suggests the presence of asymmetric volatility dynamics and heavy-tailed behavior in Brent oil returns. Accordingly, the gjrGARCH specification was selected as the preferred volatility model for subsequent estimation and forecasting analysis.

To compare the forecasting performance of the benchmark ARIMAX model and the selected ARIMAX-gjrGARCH specification, a rolling-origin forecasting framework was employed under 1-step, 3-step, 6-step, and 9-step forecast setups. Forecast accuracy was evaluated using symmetric mean absolute percentage error (sMAPE).

The forecasting results reported in Table 5, Table 6 and Table 7 provide several important insights regarding the comparative performance of statistical and machine learning approaches under alternative train-test split scenarios.

First, the findings consistently demonstrate that LightGBM emerged as the most competitive forecasting model across the majority of forecasting horizons and data partitions. Under the primary 80-20% split presented in Table 5, LightGBM achieved the lowest sMAPE values in nearly all rolling forecast settings, including both short-term (1-step, 3-step) and longer horizons (6-step and 9-step).

This superior performance is not limited to a single data partition. As shown in Table 6 and Table 7, the results remained highly consistent under the alternative 75-25% and 70-30% splits. Across these robustness scenarios, LightGBM continued to outperform competing models, indicating that its predictive advantage is stable and not driven by sample-specific characteristics.

These findings suggest that the relationship between Brent oil returns and the selected predictors is governed by nonlinear interactions and complex temporal dependencies, which are more effectively captured by gradient boosting frameworks such as LightGBM.

The performance of the ARIMAX model remains noteworthy. As reported in Table 5, Table 6 and Table 7, ARIMAX occasionally achieved competitive results in selected horizons, particularly in medium- and long-term forecasts. This outcome is theoretically consistent, as oil return series often exhibit short-run autoregressive structures that can be effectively captured by linear lag-based models.

Nevertheless, the overall evidence indicates that machine learning models, particularly LightGBM, provide more robust and consistently lower forecast errors across alternative horizons, highlighting the limitations of purely linear specifications in capturing complex market dynamics.

In contrast, the ARIMAX-gjrGARCH specification does not provide superior forecast accuracy, despite its theoretical advantage in modeling conditional volatility clustering. As observed consistently across Table 5, Table 6 and Table 7, the ARIMAX-gjrGARCH model yielded higher sMAPE values than both ARIMAX and LightGBM across nearly all forecasting horizons. This result should be interpreted in light of the primary function of GARCH-type models. Although the ARIMAX-gjrGARCH specification successfully captures volatility clustering and time-varying uncertainty in Brent oil returns, its contribution to improving point forecasts of the conditional mean is inherently limited. This is because GARCH models are designed to model second-moment dynamics (conditional variance) rather than first-moment dynamics (conditional mean).

Therefore, the relatively weaker performance of ARIMAX-gjrGARCH in terms of sMAPE does not indicate model inadequacy, but rather reflects the distinction between volatility modeling and mean forecasting. In contrast, machine learning models are directly optimized to minimize prediction error in the target variable, enabling them to better capture nonlinear patterns affecting the conditional mean of returns.

The random forest model produced comparatively weaker forecasting performance across most scenarios. As shown in Table 5, Table 6 and Table 7, its sMAPE values were generally higher than those of LightGBM, and in many cases, also higher than XGBoost. This comparatively weaker performance may indicate that the oil return dynamics require a modeling framework capable of capturing more refined nonlinear structures, interaction effects, and gradient-based adjustments, which are more effectively handled by boosting algorithms.

The strong performance of LightGBM can be attributed to its ability to efficiently model nonlinear relationships, handle feature interactions, and adapt to varying data structures through gradient-based optimization. Compared to alternative machine learning models, LightGBM provides a more effective balance between model flexibility, regularization, and generalization capability within the present forecasting framework.

Consequently, the empirical evidence presented in Table 5, Table 6 and Table 7 strongly supports the view that machine learning models, particularly LightGBM, provide substantial forecasting advantages in modeling Brent oil return dynamics under strategic risk conditions. At the same time, the competitive performance of ARIMAX in selected horizons confirms that linear benchmark models continue to offer valuable interpretability and baseline predictive insights. From an information-theoretic perspective, the superior performance of LightGBM suggests that tree-based boosting algorithms more effectively exploit nonlinear information embedded in lagged returns and strategic risk indicators. This finding reinforces the view that Brent oil return dynamics are governed by complex, nonlinear information structures shaped by financial and geopolitical uncertainty.

Table 8 reports the Diebold-Mariano (DM) test results based on sMAPE loss. The null hypothesis assumes equal forecast accuracy between models. Negative DM statistics indicate that the first model performed better than the second. Statistically significant *p*-values (*p* < 0.05) suggest that the difference in forecasting performance is meaningful. The results confirm that LightGBM significantly outperforms XGBoost, ARIMAX, random forest, and ARIMAX-gjrGARCH in terms of forecast accuracy, further supporting its superiority in modeling nonlinear oil return dynamics. In addition, the comparison between ARIMAX and ARIMAX-gjrGARCH indicates no statistically significant difference in forecast accuracy (*p* > 0.05), suggesting that incorporating conditional volatility does not lead to a meaningful improvement in point forecast performance.

**Figure 8:**
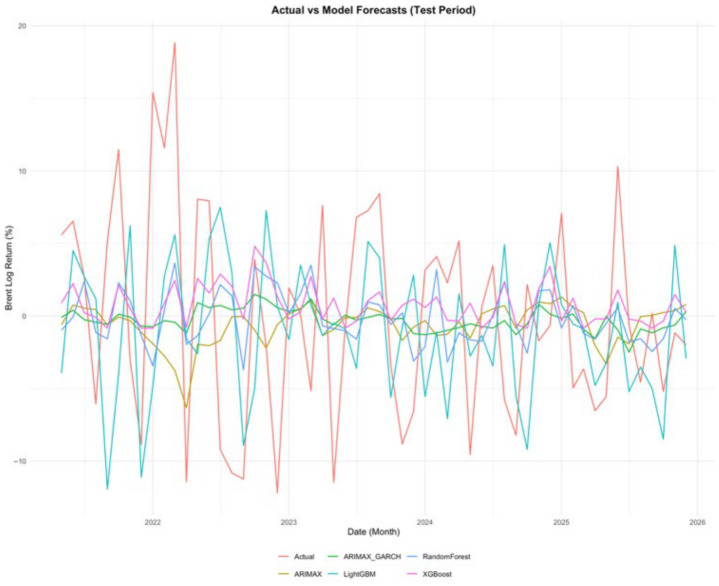
Comparison of actual brent returns and alternative model forecasts in the test period under the 80-20% train-test split.

Figure 8 presents the actual Brent log returns (red line) together with the forecasts generated by the ARIMAX (gold), ARIMAX-gjrGARCH (green), XGBoost (magenta), random forest (blue), and LightGBM (cyan) models over the test period under the 80-20% train-test split. The figure provides a visual comparison of how closely each model follows the observed movements in Brent returns, particularly in terms of directional changes, local fluctuations, and short-run volatility patterns. While all models capture certain aspects of the return dynamics, the degree of alignment with the actual series differs across models.

A visual analysis of Figure 8 indicates that the LightGBM model tracks short-term fluctuations and local turning points more closely than the competing models. Its forecasts exhibit a higher degree of responsiveness to rapid changes in the return series, suggesting an enhanced ability to capture nonlinear and time-varying dynamics. In contrast, the ARIMAX and ARIMAX-gjrGARCH models produce comparatively smoother trajectories, reflecting their reliance on linear structures and conditional volatility modeling, respectively. The Random Forest and XGBoost models display intermediate behavior, capturing some nonlinear patterns but with less consistent alignment to local movements.

However, this visual representation should be interpreted cautiously and in conjunction with the quantitative forecast accuracy measures reported in Table 5, Table 6 and Table 7. While LightGBM forecasts exhibit a closer visual resemblance to the observed series, the primary evidence of its superiority lies in its consistently lower sMAPE values across alternative forecasting horizons and train–test splits.

Accordingly, Figure 8 supports the broader empirical findings by illustrating that LightGBM not only captures short-term fluctuations more effectively in a visual sense but also achieves superior predictive accuracy in terms of forecast error metrics. This consistency reinforces the conclusion that gradient boosting models provide a more suitable framework for modeling the nonlinear dynamics of Brent oil returns.

As far as Table 9 is concerned, the feature importance analysis reveals that both autoregressive dynamics and exogenous risk indicators contribute to the predictive performance of the LightGBM model. In this context, gain measures the contribution of each feature to the model by quantifying the improvement in prediction accuracy brought by splits on that variable; cover reflects the relative number of observations affected by the feature; and frequency indicates how often the feature is used in the tree-building process. Among all variables, the first lag of Brent returns (lag1) had the highest contribution, followed closely by the lagged Geopolitical Risk Index (GPR), indicating that geopolitical risk is a key driver of oil return predictability.

While financial market uncertainty (VIX) and interest rate changes (ΔDGS10) also contribute to the model, their relative importance is more moderate. The entropy-based variable exhibits a smaller but non-negligible contribution, suggesting that it provides complementary information rather than serving as a primary predictive driver.

Thus, these findings confirm that the model captures both internal market dynamics and external risk signals, supporting the relevance of strategic risk indicators in oil price forecasting.

As reported in Table 10, the ablation analysis provides clear evidence on the incremental contribution of different feature groups to the forecasting performance of the LightGBM model. When only lagged Brent returns were used as predictors, the model yielded an sMAPE of 1.4902. The inclusion of strategic risk variables (GPR, VIX, and DGS10) led to a noticeable improvement, reducing the sMAPE to 1.4789. This finding indicates that macro-financial and geopolitical risk indicators contain additional predictive information beyond the internal dynamics of the series. Furthermore, augmenting the model with the entropy-based variable resulted in a further, albeit modest, improvement in performance, with sMAPE decreasing to 1.4718. Overall, these results suggest that both strategic risk indicators and entropy contribute positively to forecast accuracy, with the majority of the performance gain arising from the inclusion of risk-related variables. These improvements are also consistent with the Diebold–Mariano test results, which indicate that the performance differences are statistically meaningful.

## 5. Conclusions

This study examines the forecasting performance of benchmark econometric models and machine learning techniques in predicting Brent crude oil returns under strategic risk conditions. The empirical findings consistently demonstrate that machine learning approaches, particularly LightGBM, provide superior out-of-sample forecasting accuracy across multiple rolling forecast horizons and alternative train-test split configurations. The results indicate that incorporating geopolitical risk (GPR), financial market uncertainty (VIX), and macro-financial indicators (DGS10) as exogenous variables significantly enhances predictive performance.

The results indicate that the dynamics of Brent oil returns are affected by complex and nonlinear interactions among market uncertainty, geopolitical developments, and macroeconomic conditions. The superior performance of LightGBM highlights the importance of flexible, nonlinear modeling frameworks in capturing these interactions more effectively than traditional linear econometric models. While ARIMAX remains a competitive benchmark in certain forecast horizons, its performance is generally surpassed by machine learning models, indicating the presence of nonlinear dependencies not fully captured by linear specifications.

At the same time, the results emphasize an important methodological distinction between volatility modeling and return forecasting. While the ARIMAX–gjrGARCH framework provides a more realistic representation of time-varying volatility and uncertainty through conditional heteroskedasticity, its contribution to improving point forecast accuracy remains limited. This reflects the fundamental design of GARCH-type models, which focus on second-moment dynamics (conditional variance) rather than directly enhancing predictions of the conditional mean. In contrast, machine learning models—especially LightGBM—are explicitly optimized to minimize prediction error, enabling them to better capture nonlinear relationships and interaction effects in return dynamics.

From an economic perspective, these findings have important implications for energy markets and risk management practices. The strong predictive contribution of financial uncertainty variables implies that oil return dynamics are highly sensitive to shifts in global risk perception and macro-financial conditions. Market participants including commodity traders, institutional investors, and energy firms may benefit from integrating such indicators into their forecasting, hedging, and risk assessment frameworks. More accurate return forecasts can support improved value-at-risk (VaR) estimation, derivative pricing, and dynamic hedging strategies.

From a policy perspective, the findings suggest that policymakers and energy authorities should meticulously observe strategic risk indicators when evaluating potential energy price shocks. Given the rapid transmission of oil price fluctuations into inflation, production costs, and macroeconomic expectations, enhanced forecasting frameworks can support more proactive and informed policy responses. In particular, central banks, energy ministries, and regulatory institutions may incorporate such models into early-warning systems to detect adverse market developments and mitigate macroeconomic risks.

Furthermore, the findings have broader implications for understanding the transmission of energy price shocks across the real economy. Due to crude oil’s irreplaceable role for transportation, manufacturing, logistics, and electricity generation in many economies, fluctuations in oil returns can rapidly influence production costs, consumer prices, and inflation expectations. In this context, more accurate oil return forecasting is not only a financial market effort but also an important tool for predicting broader macroeconomic pressures. The superior predictive performance of machine learning models therefore suggests that policymakers and market analysts may improve shock preparedness by relying on forecasting frameworks that better capture nonlinear market reactions during periods of elevated uncertainty.

The results are also relevant for energy-dependent firms and importing economies, where exposure to oil price volatility directly affects budgeting, procurement, and strategic planning decisions. Airlines, logistics organizations, petrochemical companies, and energy-intensive manufacturers may use such forecasting evidence to refine fuel cost estimations and optimize hedging or contracting strategies. Likewise, for net oil-importing countries, more reliable forecasts can support decisions related to foreign exchange planning, subsidy design, and contingency measures to external energy shocks. In this sense, the forecasting framework proposed in this study may contribute not only to financial optimization but also to operational resilience and policy coordination.

From a broader academic and policy standpoint, the study also highlights the need to move beyond traditional forecasting structures when examining commodity markets under strategic risk conditions. The fact that LightGBM consistently outperforms the benchmark models indicates that the predictive content of geopolitical and financial uncertainty is embedded in relationships that may be nonlinear, state-dependent, and interaction-driven. This has an important implication for future research in energy economics: model formulation should progressively incorporate asymmetry, instability, and nonlinear transmission channels instead of assuming uniform responses across all market conditions. Accordingly, the study contributes to both the forecasting literature and the policy debate by showing that more flexible data-driven approaches can provide stronger empirical guidance in environments characterized by uncertainty, volatility, and strategic risk.

Moreover, the evidence that financial uncertainty plays a dominant role in oil return forecasting suggests that energy policy decisions must be assessed in conjunction with financial market conditions. Energy security policies, strategic reserve planning, and price stabilization measures may benefit from integrating financial risk indicators into decision-making processes.

Collectively, this study contributes to the growing literature on energy forecasting by demonstrating that machine learning techniques provide substantial advantages in modeling oil return dynamics under strategic risk conditions. Future research may extend this framework by incorporating higher-frequency data, regime-switching structures, or deep learning architectures to further improve predictive performance and policy relevance. Furthermore, the analysis incorporates an entropy-based measure to capture the informational complexity and uncertainty structure of Brent oil returns. While the inclusion of entropy provides additional insights into the evolving dynamics of uncertainty, its contribution to improving point forecast accuracy remains limited. This suggests that entropy captures aspects of market complexity that are not directly exploitable for short-term return prediction but may still be valuable for understanding uncertainty transmission and informational structure in energy markets.

Overall, the findings suggest that combining traditional econometric approaches with machine learning techniques and information-theoretic measures provides a more comprehensive framework for analyzing oil market dynamics under uncertainty. While machine learning models excel in predictive accuracy, econometric and entropy-based approaches contribute to interpretability and a deeper understanding of underlying market mechanisms.

## Figures and Tables

**Table 1:** Descriptive statistics of the variables used in the study.

Variable	Mean	SD	Min	Max
Brent return	0.29	10.21	−55.41	46.91
GPR	11,030.91	5123.86	5057.00	51,253.00
VIX	19.68	8.08	10.13	62.67
DGS10	3.21	1.19	0.62	5.39

**Table 2:** Unit root test results.

Variable	ADF Statistic	ADF *p*-Value	PP Statistic	PP *p*-Value	Conclusion
Brent return	−6.586	0.01	−182.46	0.01	Stationary
GPR	−3.604	0.03	−65.39	0.01	Stationary
VIX	−3.591	0.03	−44.45	0.01	Stationary
DGS10	−1.376	0.84	−6.580	0.741	Non-stationary
DGS10 (∆)	−7.176	0.01	−190.82	0.01	Stationary

**Table 3:** Correlation matrix of the variables.

Variable	Brent Return	GPR	VIX	DGS10
Brent return	1.000	−0.070	−0.261	0.033
GPR	−0.070	1.000	0.110	0.303
VIX	−0.261	0.110	1.000	−0.057
DGS10	0.033	0.303	−0.057	1.000

**Table 4:** Model selection results for alternative GARCH specifications with a skewed Student’s *t* distribution.

Model	AIC	BIC	LogLik
sGARCH	7.0562	7.2408	−676.9778
eGARCH	7.0442	7.2624	−673.8147
**gjrGARCH**	**7.0171**	**7.2521**	**−670.1651**

**Table 5:** Forecast accuracy by rolling step using the 80-20% training-test set.

	Horizon	LightGBM	XGBoost	ARIMAX	Random Forest	ARIMAX-gjrGARCH
1-step	1	**1.39**	1.56	1.55	1.60	1.65
	1	**1.43**	1.55	1.57	1.61	1.67
3-step	2	**1.44**	1.64	1.71	1.64	1.76
	3	**1.44**	1.59	1.71	1.62	1.77
	1	**1.47**	1.56	1.55	1.60	1.67
	2	**1.45**	1.65	1.71	1.65	1.78
6-step	3	**1.44**	1.59	1.69	1.63	1.79
	4	**1.46**	1.55	1.73	1.67	1.79
	5	**1.45**	1.43	1.73	1.64	1.77
	6	**1.42**	1.51	1.74	1.63	1.76
	1	**1.48**	1.55	1.56	1.61	1.66
	2	**1.48**	1.68	1.71	1.63	1.79
	3	**1.44**	1.60	1.72	1.63	1.81
	4	**1.50**	1.53	1.71	1.69	1.80
9-step	5	**1.43**	1.42	1.71	1.63	1.79
	6	**1.42**	1.50	1.72	1.64	1.79
	7	**1.29**	1.50	1.70	1.68	1.79
	8	**1.55**	1.58	1.72	1.68	1.77
	9	**1.42**	1.69	1.72	1.65	1.78

**Table 6:** Forecast accuracy by rolling step using the 75-25% training-test set.

	Horizon	LightGBM	XGBoost	ARIMAX	Random Forest	ARIMAX-gjrGARCH
1-step	1	**1.41**	1.53	1.49	1.58	1.57
	1	**1.42**	1.52	1.50	1.57	1.59
3-step	2	**1.50**	1.60	1.75	1.61	1.77
	3	**1.41**	1.59	1.75	1.64	1.78
	1	**1.44**	1.53	1.49	1.55	1.58
	2	**1.53**	1.60	1.75	1.63	1.79
6-step	3	**1.43**	1.60	1.74	1.65	1.79
	4	**1.32**	1.51	1.75	1.64	1.79
	5	**1.24**	1.43	1.70	1.61	1.77
	6	**1.32**	1.54	1.76	1.65	1.76
	1	**1.46**	1.52	1.49	1.54	1.57
	2	**1.56**	1.62	1.75	1.63	1.80
	3	**1.44**	1.60	1.76	1.66	1.81
	4	**1.34**	1.48	1.74	1.65	1.80
9-step	5	**1.22**	1.43	1.69	1.63	1.79
	6	**1.34**	1.53	1.75	1.66	1.79
	7	**1.37**	1.50	1.73	1.66	1.81
	8	**1.34**	1.53	1.73	1.66	1.80
	9	**1.29**	1.64	1.75	1.61	1.79

**Table 7:** Forecast accuracy by rolling step using the 70-30% training-test set.

	Horizon	LightGBM	XGBoost	ARIMAX	Random Forest	ARIMAX-gjrGARCH
1-step	1	**1.41**	1.49	1.50	1.56	1.59
	1	**1.42**	1.45	1.51	1.54	1.60
3-step	2	**1.49**	1.53	1.71	1.60	1.77
	3	**1.45**	1.51	1.73	1.59	1.78
	1	**1.44**	1.48	1.50	1.56	1.59
	2	**1.52**	1.48	1.71	1.63	1.79
6-step	3	**1.49**	1.48	1.72	1.65	1.80
	4	**1.44**	1.53	1.74	1.70	1.81
	5	**1.35**	1.55	1.73	1.66	1.80
	6	**1.42**	1.53	1.76	1.68	1.78
	1	**1.46**	1.45	1.50	1.55	1.59
	2	**1.54**	1.51	1.71	1.63	1.79
	3	**1.50**	1.54	1.74	1.62	1.81
	4	**1.47**	1.54	1.73	1.69	1.81
9-step	5	**1.35**	1.46	1.72	1.62	1.81
	6	**1.44**	1.49	1.75	1.67	1.81
	7	**1.40**	1.56	1.74	1.69	1.81
	8	**1.39**	1.59	1.74	1.68	1.80
	9	**1.39**	1.57	1.73	1.65	1.80

**Table 8:** Diebold-Mariano test results for forecast accuracy comparison (80-20% split, 1-step ahead).

Comparison	DM_Statistic	*p*-Value
LightGBM vs. XGBoost	−2.6154	0.005739
LightGBM vs. ARIMAX	−2.3963	0.009994
LightGBM vs. Random Forest	−1.9290	0.029447
LightGBM vs. ARIMAX-gjrGARCH	−3.5324	0.000421
ARIMAX vs. ARIMAX-gjrGARCH	−1.4476	0.076698

**Table 9:** Feature importance ranking of the LightGBM model (80-20% train–test split).

Feature	Gain	Cover	Frequency
Lag1	0.1160	0.0820	0.0827
GPR	0.1085	0.1036	0.1014
Lag4	0.1007	0.1072	0.1053
Lag6	0.0922	0.1057	0.1023
Lag7	0.0915	0.0999	0.1013
Lag8	0.0864	0.0883	0.0866
Lag3	0.0827	0.0862	0.0906
VIX	0.0789	0.0809	0.0778
Lag5	0.0734	0.0724	0.0699
Lag2	0.0721	0.0655	0.0679
ΔDGS10	0.0643	0.0708	0.0739
Entropy	0.0333	0.0376	0.0403

**Table 10:** Ablation analysis of the LightGBM model under alternative feature sets (80-20% train–test split).

Model	sMAPE
Only lags	1.4902
Lags + Strategic Risk Variables	1.4789
Lags + Strategic Risk Variables + Entropy	1.4718

## Data Availability

The data used in this study are publicly available from open-access sources. Brent crude oil prices (DCOILBRENTEU), the CBOE Volatility Index (VIXCLS), and the U.S. 10-year Treasury yield (DGS10) were obtained from the Federal Reserve Economic Data (FRED) database. The Global Geopolitical Risk Index (GPR) was obtained from the publicly available dataset provided by Caldara and Iacoviello. All corresponding source links are provided in the References section.

## Abbreviations

The following abbreviations are used in this manuscript:

GPR	Geopolitical Risk Index
VIX	CBOE Volatility Index
DGS10	U.S. 10-year Treasury Index
ARIMAX	Auto-Regressive Integrated Moving Average with eXogenous factors
ML	Machine Learning
DL	Deep Learning
XGBoost	Extreme Gradient Boosting
LightGBM	Light Gradient Boosting Machine
LSTM	Long Short-Term Memory
ANFIS	Adaptive Neuro-Fuzzy Inference System
EPU	Emergency Power Unit
GARCH	Generalized Autoregressive Conditional Heteroskedasticity
SVR	Support Vector Regression
FRED	Federal Reserve Economic Data
ARCH	Autoregressive Conditional Heteroskedasticity
sMAPE	symmetric Mean Absolute Percentage Error
ADF	Augmented Dickey-Fuller
PP	Phillips-Perron
ACF	Autocorrelation Function
PACF	Partial Autocorrelation Function
ARIMA	Auto-Regressive Integrated Moving Average
Q–Q plot	Quantile-Quantile plot
CUSUM	Cumulative Sum
MOSUM	Moving Sum of Recursive Residuals
AIC	Akaike Information Criterion
BIC	Bayesian Information Criterion
sGARCH	Symmetric GARCH
eGARCH	Exponential GARCH
gjrGARCH	Glosten-Jagannathan-Runkle GARCH
VaR	Value-at-Risk
